# Repurposed therapeutic agents targeting the Ebola virus: a protocol for a systematic review

**DOI:** 10.1186/s13643-015-0153-9

**Published:** 2015-11-25

**Authors:** Hussein Sweiti, Obinna Ekwunife, Thomas Jaschinski, Stefan K. Lhachimi

**Affiliations:** Public Health, University Hospital Düsseldorf, Düsseldorf, Germany; Surgical Department, Klinikum Frankfurt Höchst, Frankfurt, Germany; Collaborative Research Group for Evidence-Based Public Health, Department of Prevention and Evaluation, Leibniz Institute for Prevention Research and Epidemiology - BIPS, Bremen, Germany; Department of Clinical Pharmacy and Pharmacy Management, Nnamdi Azikiwe University, Awka, Nigeria; Department for Evidence-based health services research, Institute for Research in Operative Medicine, Witten/Herdecke University, Witten, Germany; Institute for Public Health, Health Sciences Bremen, University of Bremen, Bremen, Germany

**Keywords:** Ebola virus, Ebola virus disease, Ebola haemorrhagic fever, Pharmacotherapy, Drug repurposing, Anti-Ebola drugs, Systematic review, Proposal

## Abstract

**Background:**

The recent Ebola epidemic in western Africa developed into an acute public health emergency of unprecedented level in modern times. The treatment provided in most cases has been limited to supportive care, as no approved therapies are available to date. Several established, licenced drugs have been suggested as potential repurposed therapeutic agents for Ebola. However, scientific data on their efficacy in treating Ebola is limited. The purpose of this review is to systematically assess scientific evidence on potential drugs targeting Ebola. In specific, we aim to (1) identify drug library screens involving therapeutic agents targeting the Ebola virus, (2) list potential approved drugs identified from drug screens and review their mechanism of action against the Ebola virus and (3) summarise the outcome of preclinical and clinical trials investigating approved drugs targeting the Ebola virus.

**Methods/design:**

We will develop comprehensive systematic search strategies and will perform a systematic literature search in MEDLINE, Embase and Cochrane Central Register of Controlled Trials (CENTRAL). Two authors will independently screen the titles, abstracts and the references of all selected articles on the basis of inclusion criteria. These include any available drug screening, preclinical studies and clinical studies examining the efficacy of approved therapeutic agents targeting the Ebola virus. There will be no restrictions on the type of participants, the type of comparator, time or setting. Data extraction and quality assessment will be undertaken by two review authors working independently.

**Discussion:**

This systematic review will provide systematic knowledge on potential repurposed therapeutic agents targeting Ebola. It aims to help guide future investigations on repurposed drugs and avoid repetitive studies.

**Systematic review registration:**

PROSPERO CRD42015024349

**Electronic supplementary material:**

The online version of this article (doi:10.1186/s13643-015-0153-9) contains supplementary material, which is available to authorized users.

## Background

The recent Ebola epidemic in western Africa developed into an unprecedented global public health crisis with significant humanitarian consequences. As of October 25, 2015, a total of 28,575 infected patients and 11,313 deaths have been documented worldwide [1]. The treatment provided in the great majority of cases has been limited to supportive care as no approved therapies or vaccines are available. The recommended supportive therapy includes balancing the patient’s fluids and electrolytes, maintaining the blood pressure and oxygen supply, as well as treating for any accompanying complications [2].

With a case fatality rate for the Ebola virus disease (EVD) averaging 50 % [2], there is increasing pressure to develop targeted therapeutic agents. This has also triggered an intensive debate on the use of drug repurposing in Ebola [3]. The concept of drug repurposing, also known as drug repositioning, refers to the application of established drugs in novel therapeutic indications that have not been previously approved [4]. In addition to fast-tracking the development of anti-Ebola drugs, many scientists have endorsed the clinical use of repurposed drugs to treat EVD [3, 5, 6].

Various readily available drugs have been examined as potential therapeutic agents targeting the Ebola virus life cycle or the associated immune reaction. These include blood transfusions from EVD survivors, antimalarial drug chloroquine and antiarrhythmic agents such as amiodarone, amongst many others [6]. These therapeutic agents have already passed essential toxicity and safety tests and can bypass phase I and phase IIa clinical trials. In addition, previous clinical data and experience provide valuable information on the drug’s pharmacokinetic behaviour and long-term toxicity [4]. The presence of an established manufacturing and distribution networks for these drugs is of particular importance as it allows for rapid availability in urgent cases [7]. Nonetheless, scientific evidence on the efficacy of various repurposed drugs in treating Ebola is limited, and additional investigations to justify their use in the treatment of EVD are necessary.

On the other hand, conventional drug development requires a lengthy and costly period to establish the safety and dosage of novel drugs as well as ensuring availability in sufficient amounts. It is estimated that it takes more than 10 years and over 2 billion US dollars to develop a new pharmaceutical drug [8]. The recent Ebola outbreak has revealed that, despite a fast-track programme to accelerate the development of experimental anti-Ebola drugs and vaccines, delays at different stages of development and production may have occurred [9, 10].

So far, a variety of literature reviews have been published on therapeutic targets for EVD, some of which also include an overview of possible candidates for drug repurposing [6, 11–15]. However, no systematic review dedicated to repurposed therapeutic agents targeting Ebola exists to date. Given the lack of a systematic assessment of current evidence, we aim to systematically review current scientific evidence on potential repurposed drugs targeting Ebola.

### Objectives

The aim of this review is as follows:

1. To identify available library screens involving repurposed therapeutic agents targeting the Ebola virus. These include virtual or high throughput screens which allow researchers to identify a subset of compounds that are likely to bind to a target in the Ebola virus, such as an enzyme or a receptor.
2. To list therapeutic agents, approved by the Food and Drug Administration (FDA) or an equivalent agency, with potential anti-Ebola virus effects identified from drug screens and to report their proposed mechanism of action.
3. To summarise the outcome of preclinical and clinical trials investigating repurposed drugs targeting the Ebola virus.

With our proposed systematic review, we hope to provide clinicians and scientists with an overview of scientific evidence on potential repurposed drugs targeting the Ebola virus, with a particular focus on the outcome of preclinical and clinical trials. This will not only guide future investigations aiming at providing further evidence on efficacy but will also help avoid unnecessary or repeated studies.

## Methods and design

### Proposal

The proposed review protocol conforms to the Preferred Reporting Items in Systematic Reviews and Meta-analyses (PRISMA) statement and the associated checklist [16]. The protocol is registered in the International Prospective Register of Systematic Reviews PROSPERO CRD42015024349.

### Eligibility criteria

#### Study designs

The following are our inclusion criteria:

- Studies on drug library screens which yielded at least one approved therapeutic agent targeting the Ebola virus.
- Any available preclinical and clinical studies examining the efficacy of approved therapeutic agents targeting the Ebola virus.
- Preclinical trials which may include studies on cell cultures, on mouse models or non-human primates.
- Clinical trials on humans which may include randomised controlled trials (RCTs), controlled clinical trials (CCTs), prospective and retrospective comparative cohort studies, as well as case-control studies. Cross-sectional studies, case series and case reports may also be included.

#### Study population, timing and setting

There will be no restrictions on the type of participants in preclinical or clinical trials. In addition, there will be no restrictions on the type of setting. We will include studies published from 1976, the year of discovery of the Ebola virus.

#### Types of intervention

We will include interventions involving drug library screening, preclinical studies or clinical studies examining the use approved therapeutic agents targeting the Ebola virus.

#### Comparators

There will be no restrictions on the type of comparator. Placebo, supportive care or other therapeutic interventions will be accepted.

#### Outcomes

The primary outcomes will include mortality, sequelae of the infection and serious adverse events. Secondary outcomes include adverse events. Outcomes will be collected as reported. We will extract outcomes in all data forms (e.g. dichotomous, continuous) as reported in the included studies.

#### Languages

We will include articles reported in the English, German, French and Spanish languages. A list of possibly relevant titles in any other language will be provided as an appendix.

#### Publication status

We will include articles published in scientific journals as well as unpublished ones.

### Information sources

Literature search strategies will be developed using medical subject headings (MeSH) and text words related to the Ebola virus. We will perform a systematic literature search in MEDLINE, Embase and Cochrane Central Register of Controlled Trials (CENTRAL). Studies published between January 1976 and the date the searches are run will be sought.

To identify ongoing and unpublished studies, we will search the World Health Organization (WHO) International Clinical Trials Registry Platform (ICTRP), ClinicalTrials.gov and EU Clinical Trials Register. In addition, we will search the reference lists of selected studies as well as the websites of regulatory authorities (US Food and Drug Administration and European Medicine Agency).

### Search strategy

A search strategy will be developed for each of the databases with the help of an information specialist [see Additional file 1]. The database records yielded by all search strategies will be exported into EndNote, and duplicates will be removed manually. The results of our database searches and records identified from other sources will be documented and depicted in a PRISMA flow diagram.

### Study selection

Prior to formal screening, a preliminary study screening spreadsheet [see Additional file 2: Table S1] will be used by two authors (HS and OE) to carry out a pilot screening using 50 randomly chosen studies from the search results. If necessary, the study selection spreadsheet will be refined after the pilot screening. Following the pilot screening, both authors will independently screen the titles and abstracts yielded by the search against the inclusion criteria. In addition, they will screen the reference lists of all selected articles. Studies selected at title and abstract level will be further screened for eligibility by assessing the full text of the article. We will seek additional information from study authors where necessary to resolve questions about eligibility. Opinion of a third reviewer (SKL) will be sought to arrive at a consensus in case of disagreement on a study for inclusion. We will document the reasons for excluding trials at the full-text screening level. Neither of the review authors will be blind to the journal titles or to the study authors or institutions. We will report the results of the study selection process and reasons for exclusion at the full-text screening level using a PRISMA flow diagram.

### Data extraction

Initially, two authors (HS and OE) will independently extract the names and active ingredients of all drugs targeting the Ebola virus suggested by drug library screening studies. A preliminary data extraction spreadsheet [see Additional file 3: Table S2] will be used to conduct a pilot test carried out by both authors using five randomly selected papers. The data extraction sheet will be refined accordingly after the pilot studies. The refined spreadsheet will be used by both authors to independently extract data from all included preclinical and clinical trials. Opinion of a third reviewer (SKL) will be sought to arrive at a consensus in case of disagreement. Study authors will be contacted for further clarification if necessary.

Qualitative data reported in studies included in the review will be excluded from the review and thus will not be extracted. However, if an included study draws conclusion based on qualitative data, we will report those conclusions separately in the characteristics of study table.

### Data items

Following are the items we will extract from the different types of studies [see Additional file 3: Table S2].

### Study quality

The same reviewers involved in data extraction will independently evaluate the quality of eligible studies. Internal validity of preclinical studies (animal studies) will be assessed using the SYRCLE’s risk or bias tool [17]. As per the instructions outlined in the Cochrane Handbook for systematic reviews of interventions, the Newcastle-Ottawa scale will be used to assess quality of non-randomised studies. If an adequate number of randomised controlled trials are identified in our search, the Cochrane Collaboration’s tool for assessing risk of bias will be used.

### Statistical analysis

We expect studies to be too heterogeneous to allow for a quantitative summary of results. If, however, we find subsets of studies to be homogeneous enough, we will perform a random effects meta-analysis for all primary outcomes. Heterogeneity will be detected through visual inspection of the forest plots and by using a standard chi^2^ test with a significance level of *P* < 0.10. The *I*^2^ statistic will be applied to quantify inconsistency across studies and to assess the impact of heterogeneity on the meta-analysis. *I*^2^ ≥ 50 % indicates substantial heterogeneity.

Subgroup analyses will be conducted on the primary outcomes, whether meta-analysed or synthesised narratively, if feasible. Subgroups investigated will be at least age, sex and region.

Studies with separate control groups are likely to measure the effect of treatment on a dichotomous or ordinal health outcome as a risk ratio, odds ratio or risk difference between the treatment and control groups. This review will prioritise risk ratios. In cases where a risk ratio is not reported in a record, we will either calculate it from data reported in the record or request a risk ratio measure or the data required to calculate it from the principal author of the record, contacting them by email or phone, using the contact details provided in the record. When included studies report treatment effects on continuous outcomes as mean differences between treatment and control groups, we will report the mean difference. Where different scales are used for the same outcome, the review will report the standardised mean difference (Hedges’ g).

If a study presents adjusted and unadjusted treatment effect measures, the review will report the adjusted measures. If only unadjusted treatment effect measures are provided, but measures for between-group differences in covariates at baseline and/or potential confounders are also reported, then we will adjust the treatment effect measure for these variables. However, if the required information for adjustment of treatment effect estimates is not provided, we will request from the principal study author by email or phone an adjusted treatment effect estimate. If we are only able to obtain unadjusted treatment effect measures, we will report and use these measures, with the appropriate caveats.

Intention-to-treat treatment effect measures will be prioritised over other treatment effect measures. If feasible, 95 % confidence intervals will be provided for each treatment effect measures.

## Discussion

The recent Ebola outbreak is an unprecedented public health risk with a high mortality rate and no approved targeted treatment to date. Repurposed therapeutic agents represent a valid approach to finding a treatment for EVD, and they offer significant advantages in terms of saving time and financial resources. However, additional scientific evidence on their efficacy is essential to justify their use in the treatment of EVD.

This systematic review will give an overview of the existing data on repurposed therapeutic agents targeting Ebola. It will assess the current level of scientific evidence for candidate therapeutic agents from the drug screening stage to the clinical trial stage and will outline the strengths and limitations of identified evidence. Furthermore, this review will help expose areas for potential research on repurposed drugs for the treatment of EVD, hence guiding future investigations in this field. The limitations and strengths of this review will be discussed, and results from other relevant systematic reviews will be compared to the results of this review.

## Additional files

Additional file 1:
**Search strategies.** Search strategies for Medline, Embase and Central databases. (DOC 29 kb) Additional file 2: Table S1. Study screening. Study screening spreadsheet for the selection of eligible studies. (DOC 31kb) Additional file 3: Table S2. Data items. Data items for extraction from different types of selected studies. (DOC 35 kb)

## Abbreviations

EVD: Ebola virus disease
FDA: Food and Drug Administration
RCT: randomised controlled trials
CCT: controlled clinical trials
PRISMA: Preferred Reporting Items in Systematic Reviews and Meta-analyses
