# Supplementary material for: Repurposed therapeutic agents targeting the Ebola virus: a protocol for a systematic review
Source: Syst Rev. 2015 Nov 25;4:171. doi: 10.1186/s13643-015-0153-9 (PMC4658770; doi:10.1186/s13643-015-0153-9)
Supplement: Additional file 1: — Search strategies. Search strategies for Medline, Embase and Central databases. (DOC 29 kb) [file 13643_2015_153_MOESM1_ESM.doc]

Additional file 1: Search strategies

S1: Search strategy for Medline (data base) via Pubmed (search engine)

| ("Hemorrhagic Fever, Ebola"[Mesh] OR "Ebolavirus"[Mesh] OR Ebola[TIAB])  AND  ("Antiviral Agents"[Mesh] OR "Antiviral Agents" [Pharmacological Action] OR "antiviral agents"[TIAB] OR "Drug Therapy"[Mesh] OR “drug therapy”[TIAB] OR "therapeutic use" [Subheading] OR “therapeutic agents”[TIAB] OR "Therapeutics"[Mesh] OR treatment*[TIAB] OR therap*[TIAB] OR repurpos*[TIAB] OR "Drug Repositioning"[Mesh] OR (virtual[TIAB] AND screen*[TIAB])) |
| --- |

S2: Search strategy for Embase (data base) via Embase (search engine)

| ('Ebola hemorrhagic fever'/exp OR 'Ebola virus'/exp OR ebola:ab,ti)  AND  ('antivirus agent'/exp OR ‘antiviral agents’:ab,ti OR 'drug therapy'/exp OR 'drug therapy':ab,ti OR ‘therapeutic agents’:ab,ti OR 'therapy'/exp OR treatment*:ab,ti OR therap*:ab,ti OR repurpos*:ab,ti OR 'drug repositioning'/exp OR (virtual NEAR/1 screen*):ab,ti) |
| --- |

S3 Search strategy for Central (data base) via Cochrane Library (search engine)

| (MeSH descriptor: [Hemorrhagic Fever, Ebola] explode all trees OR MeSH descriptor: [Ebolavirus] explode all trees OR ebola:ti,ab,kw) AND (in Trials) |
| --- |
