# Supplementary material for: Repurposed therapeutic agents targeting the Ebola virus: a protocol for a systematic review
Source: Syst Rev. 2015 Nov 25;4:171. doi: 10.1186/s13643-015-0153-9 (PMC4658770; doi:10.1186/s13643-015-0153-9)
Supplement: Additional file 2: Table S1. — Study screening. Study screening spreadsheet for the selection of eligible studies. (DOC 31kb) [file 13643_2015_153_MOESM2_ESM.doc]

Additional file 2: Table S1: Study screening

| Study Nr. | Study Title | Reviewer #1 (HS) | | | | Reviewer #2 (OE) | | | | Comments |
| --- | --- | --- | --- | --- | --- | --- | --- | --- | --- | --- |
|  |  | Title & Abstract Screening | Full text Screening | Reason for Exclusion | References screening | Title & Abstract Screening | Full text Screening | Reason for Exclusion | References screening |  |
|  |  |  |  |  |  |  |  |  |  |  |
|  |  |  |  |  |  |  |  |  |  |  |
|  |  |  |  |  |  |  |  |  |  |  |
|  |  |  |  |  |  |  |  |  |  |  |
|  |  |  |  |  |  |  |  |  |  |  |
