# Supplementary material for: Repurposed therapeutic agents targeting the Ebola virus: a protocol for a systematic review
Source: Syst Rev. 2015 Nov 25;4:171. doi: 10.1186/s13643-015-0153-9 (PMC4658770; doi:10.1186/s13643-015-0153-9)
Supplement: Additional file 3: Table S2. — Data items. Data items for extraction from different types of selected studies. (DOC 35 kb) [file 13643_2015_153_MOESM3_ESM.doc]

Additional file 3: Table S2: Data items

| ***Drug Library Screening Studies*** | |
| --- | --- |
| Publication details | Year of publication, first author, country in which study was conducted |
| Molecular library used | e.g. Drug bank |
| Drug repurposing screen criterion | e.g. EIIP/AQVN |
| Approved compounds identified through drug screen | Drug name, active ingredient, approving drug agency |
| ***Preclinical Studies*** | |
| Publication details | Year of publication, first author, country in which study was conducted |
| Study design characteristics | Experimental groups, number of animals |
| Animal model characteristics | Species, gender, disease induction |
| Intervention characteristics | Intervention, timing, duration |
| Outcome measures | Lethal dose 50 (LD 50), effective dose 50 (ED 50), inhibitory concentration 50 (IC 50) |
| Other | Drop-outs |
| ***Clinical Studies*** | |
| Publication Details | Year of publication, first author, country in which study was conducted |
| Design | Study design, duration of study, follow-up time |
| Population Data | Population size, gender |
| Intervention | Therapeutic agent tested, dosage, frequency and duration of treatment, comparator |
| Outcome | Mortality rate, sequelae of infection, serious adverse events, immediate cause of mortality |
